# Cross-species single-cell transcriptomic analyses reveal evolutionary conservation and diversification of ovarian tissues

**DOI:** 10.1186/s40104-026-01383-1

**Published:** 2026-04-14

**Authors:** Bailing Xue, Yajing Liu, Chen Zhou, Kairat Dossybayev, Narantuya Baatar, Nikolay Yudin, Linwei Zhang, Ji Yang, Menghua Li, Songsong Xu

**Affiliations:** 1 Frontiers Science Center for Molecular Design Breeding (MOE); State Key Laboratory of Animal Biotech Breeding; College of Animal Science and Technology, China Agricultural University, Beijing, 100193 China; 2 LPP “Kazakh Research Institute for Livestock and Fodder Production”, Almaty, 050035 Kazakhstan; 3 Research Institute of Animal Husbandry, Ulaanbaatar, 17029 Mongolia; 4 Institute of Cytology and Genetics, Siberian Branch of Russian Academy of Sciences (ICG SB RAS), Novosibirsk, 630090 Russia; 5 Department of Neurology, China-Japan Friendship Hospital, Beijing, 100029 China

**Keywords:** Cross-species analysis, Evolution, Ovary, Single-cell RNA-seq, Vertebrates

## Abstract

**Background:**

The ovary is a central female reproductive organ responsible for producing oocytes and secreting steroid hormones. To investigate the molecular similarities and potential evolutionary origins of the ovary across vertebrates, we integrated publicly available single-cell and single-nucleus transcriptomic data from nine species, including oviparous animals (fish and chicken) and viviparous mammals (mouse, rat, sheep, goat, yak, monkey, and human).

**Results:**

We generated a multi-species ovarian atlas comprising 186,748 cells and identified nine cell types. Cross-species comparative analysis identified conserved and species-specific features in granulosa cells (GCs) and stromal cells (SCs), including gene expression patterns, signaling pathways, and cell–cell communication networks. For example, proliferation-associated genes such as *CKAP5*, *MCM3*, and *GINS1* were shared across vertebrates in GC-3, highlighting the evolutionary conservation of the molecular machinery underlying granulosa cell division. A comparison of granulosa cell subtypes further revealed species-specific gene co-expression patterns in oviparous and viviparous species, indicating adaptations for their different reproductive strategies. Stromal cells showed strong evolutionary conservation, as core extracellular matrix genes (e.g., *COL6A1*, *FSTL1*, *ACTN1*) were co-expressed across species in SC-1. Moreover, the conserved ligand-receptor pairs such as COL4A1-CD44, COL1A1-SDC4, LAMB1-ITGA6-ITGB1, and VEGFA-NRP2 constituted fundamental signaling axes of follicle development, while interactions such as PPIA-BSG in mouse and MDK-NCL in human were higher in these species compared with others, reflecting species-specific ovarian cell communication during evolution.

**Conclusions:**

These findings provide comprehensive insights into the molecular evolution and cellular diversity of the ovary, offering a valuable resource for understanding ovarian biology and evolutionary mechanisms.

**Supplementary Information:**

The online version contains supplementary material available at 10.1186/s40104-026-01383-1.

## Introduction

The ovary is a key reproductive organ that governs gamete maturation and hormone secretion, ensuring successful reproduction in vertebrates [1]. Despite these shared functions, ovarian structure, cellular composition, and regulatory mechanisms vary widely among species. This difference reflects distinct reproductive strategies, lineage-specific evolutionary histories, and developmental programs [2]. One of the major evolutionary transitions in vertebrate reproduction is the shift from oviparity to viviparity. In oviparous species such as fish and birds, ovarian function is primarily optimized for rapid oocyte growth and yolk accumulation to support external embryonic development. In contrast, viviparous mammals have evolved complex follicular dynamics, ovulation control, and luteal formation to sustain internal fertilization and pregnancy [3, 4]. Comparative anatomical and histological studies further suggest that several core ovarian cell types, such as granulosa, theca, stromal, and endothelial cells, are broadly conserved across vertebrates [5, 6]. However, whether this apparent conservation extends to the molecular level, and how gene regulatory programs and intercellular interactions have diversified during vertebrate evolution, remain unresolved questions.

Evolutionary conservation in ovarian biology is reflected in shared cell-type identities and orthologous gene expression, while divergence is manifested as species-specific regulatory programs or changes in cell-type composition [7, 8]. Molecular conservation is quantitatively defined as orthologous genes exhibiting similar expression patterns across species within corresponding cell types, assessed by correlation metrics and overlap of marker genes [9, 10]. Divergence is quantified as species-specific gene expression changes, differences in cell-type proportions, or unique regulatory interactions, identified through differential expression analysis and intercellular communication network comparisons [11]. However, traditional bulk transcriptomic approaches lack the resolution to capture these features in heterogeneous ovarian tissue. Single-cell and single-nucleus RNA sequencing (sc/snRNA-seq) enables detailed profiling of ovarian cell populations, capturing heterogeneity and key regulatory pathways [12–14]. Furthermore, most studies examine only single species, making it difficult to identify conserved and species-specific programs.

In this study, we used publicly available sc/snRNA-seq data to perform a cross-species analysis of ovaries from nine vertebrate species, covering major evolutionary and reproductive transitions from oviparity to viviparity. We aimed to test the hypothesis that viviparous and oviparous animals exhibit differences in ovarian cell composition and gene expression patterns. By constructing a comprehensive single-cell and single-nucleus transcriptomic atlas, we sought to uncover the conserved and divergent features of ovarian cell types, particularly granulosa and stromal cells. Specifically, we examined differences in cellular composition, conserved and species-specific gene expression programs, and the evolution of intercellular communication networks across species. Our study provides a systematic framework for understanding the molecular evolution, functional specialization, and adaptive diversity of ovarian cell types across vertebrates, offering insights into the evolution of reproductive functions.

## Materials and methods

### Sc/snRNA-seq data analysis

In this study, we collected and analyzed sequencing data from 21 ovarian samples from nine different species (Tables S1 and S2). A scRNA-seq dataset from the ovarian samples of six Chinese tongue soles and a snRNA-seq dataset from the ovary of one cynomolgus monkey were retrieved from the China National GeneBank (https://db.cngb.org/cnsa/) under accession numbers CNP0002319 [15] and CNP0001469 [16], respectively. Ovarian scRNA-seq datasets from three mice were downloaded from ArrayExpress (accession number E-MTAB-11491 [17]). scRNA-seq raw data of ovarian samples from two chickens and one rat were collected from the Genome Sequence Archive (GSA; https://ngdc.cncb.ac.cn/gsa/) under accession numbers CRA017114 [18] and CRA008987 [19], respectively. snRNA-seq datasets of ovarian samples from four goats and scRNA-seq raw data from one Dazu black goat were obtained from the NCBI GEO database (GSE207023 [20]) and NCBI BioProject (PRJNA1010653 [21]), respectively. Additionally, ovarian scRNA-seq datasets from four sheep, one yak, and three humans were accessed from the NCBI GEO database under accession numbers GSE233801 [22], GSE213989 [5], and GSE255690 [23], respectively. Furthermore, all samples included in this study were derived from adult individuals, as described in the original studies. For each species, the entire ovary was collected, and in larger species, the ovary was cut into smaller pieces (containing both cortex and medulla), preserving the overall cellular diversity of the ovary. Detailed sample information is summarized in Table S1.

### Filtering and normalization of sc/snRNA-seq data

The genome sequences and corresponding annotation files for chicken (bGalGal1.mat.broiler.GRCg7b) and rat (Rnor6.0) were downloaded from NCBI, while those for goat (ARS1) were obtained from Ensembl. Subsequently, reference genomes were constructed using the CeleScope v2.4.0 pipeline (https://github.com/singleron-RD/CeleScope) for chicken and the Cell Ranger v7.2.0 pipeline (https://github.com/10XGenomics/cellranger) for rat and goat. Reference genomes for the other species, including fish (Cse_v1.0), mouse (GRCm38), sheep (Oar_rambouillet_v1.0), yak (LU_Bosgru_v3.0), monkey (Macaca_fascicularis_5.0), and human (GRCh38), were downloaded according to the corresponding publications.

Raw sequencing data from chicken were processed using the CeleScope v2.4.0 pipeline, and the reads were aligned to the chicken reference genome to generate gene count matrices. Raw sequencing reads from rat and the Dazu black goat were processed using the Cell Ranger v7.2.0 pipeline. The reads were aligned to the rat and goat reference genomes, respectively, and gene count matrices were subsequently generated. The Seurat R package (v5.2.0; https://satijalab.org/seurat/) was used to reanalyze the datasets from all species and to perform quality control and cluster analysis. Cells or nuclei expressing at least 200 genes were retained, and genes detected in at least three cells or nuclei were retained. Based on transcriptional differences among species, cells or nuclei with excessively high gene numbers in each species or with mitochondrial gene percentages greater than 10% were also removed (Table S2). Finally, doublet removal was performed using the DoubletFinder package (v2.0.4) [24].

### Ortholog mapping and gene name conversion

To facilitate cross-species data integration, genes in each species were matched to human orthologs using the biomaRt package (v2.62.1) [25]. For genes with a one-to-one orthologous relationship, the non-human gene names were replaced with the corresponding human gene names, whereas genes without defined human orthologs were retained (Table S3). Finally, gene names from all species were converted to uppercase for consistency.

### Benchmarking integration methods

To identify the optimal integration method for our datasets, we benchmarked three widely used methods including CCA, Reciprocal principal component analysis (RPCA), and Harmony using scIB (v.1.1.7) [26]. Overall, scores were calculated as a weighted mean with weights 40:60 of batch correction and biological variance conservation, based on 10 metrics provided by scIB. Ultimately, RPCA was selected as the top-performing method for integrating the cross-species ovary datasets.

### Annotation of ovarian cell types

Cells and nuclei were normalized separately using log-normalization (scale factor = 10,000) and then clustered. The top 2,000 highly variable genes were selected using the FindVariableFeatures function with the “vst” method. Subsequently, data scaling was performed on the highly variable genes. RPCA was then applied to integrate the cross-species data using the FindIntegrationAnchors and IntegrateData functions with parameters reduction = "rpca" and dims = 1:30. Cell clustering was performed using the FindNeighbors and FindClusters functions, and dimensionality reduction was performed using RunUMAP. Marker genes for each cluster were identified using the FindAllMarkers function (logfc.threshold = 0.25, min.pct = 0.25) in Seurat.

### Cell type similarity analysis across species

MetaNeighbor (v1.26.0) [27] was used to measure the similarity between different cell types of different species. First, we analyzed all cell types of all species and then focused on granulosa and stromal cell subtypes. The similarity among cell types was assessed using the area under the receiver operating characteristic curve (AUROC) score. Highly variable genes for all cell types were identified using the variableGenes function and used as input for the MetaNeighborUS function (fast_version = TRUE). For granulosa and stromal cell subtypes, both the fast_version and one_vs_best parameters were set to TRUE, and an AUROC score above 0.9 was used as a threshold to identify highly replicable subtypes within each species and across species pairs.

### Identification of differentially expressed genes (DEGs) and functional enrichment analysis

DEGs were identified using the Seurat function FindAllMarkers. The parameters were set as follows: only.pos = TRUE, min.pct = 0.25, logfc.threshold = 0.25, and test.use = "wilcox". Each cluster was compared against all other clusters. *P* values were adjusted for multiple testing using the Bonferroni correction, and genes with adjusted *P* values < 0.05 were considered significant DEGs. Gene Ontology (GO) and Kyoto Encyclopedia of Genes and Genomes (KEGG) enrichment analyses were performed using the clusterProfiler package (v4.17.0) with the hypergeometric test. The results were visualized with the ggplot2 package (v4.0.0).

### Cross-species clustering and comparative analysis of cell subtypes

First, the RPCA method was applied to re-integrate granulosa and stromal cells, respectively. Based on marker genes, granulosa cells were further classified into six clusters, while stromal cells were divided into three clusters. For cross-species comparison, only one-to-one orthologous genes to human were retained from each of the nine species for differential gene expression analysis. The FindAllMarkers function was used to identify up-regulated genes in each cluster, with parameters consistent with those described above. According to evolutionary relationships, the nine species were categorized into five groups: fish, chicken, rodents (mouse and rat), ruminants (sheep, goat, and yak), and primates (monkey and human). Differentially expressed genes were then combined within each group. To identify conserved genes within each cluster, DEGs from the five groups were compared and their intersections were visualized using the UpSetR package. Due to the limited number of granulosa cells in the human sample, only the other eight species were retained in the analyses of granulosa cells.

### Granulosa cell downsampling and subsequent analysis

To ensure comparable numbers of granulosa cells across species, we performed six rounds of downsampling for eight species (fish, chicken, mouse, rat, sheep, goat, yak, and monkey). For species with more than 2,500 granulosa cells, excess cells were randomly removed, whereas species with fewer than 2,500 cells were retained. After reclustering, the granulosa cell subtypes obtained in each downsampling round were compared with the original subtypes to assess their correlation.

### Single-cell trajectory inference

Slingshot (v2.14.0) [28] is a computational method for inferring cell lineages and estimating gene expression dynamics along developmental trajectories. CytoTRACE was applied to assess the differentiation states of cells [29]. Using Slingshot, developmental trajectories of granulosa cell subtypes were reconstructed. The getLineages and getCurves functions were employed to infer differentiation lineages and to model dynamic changes in gene expression along pseudotime, respectively. Subsequently, the SCP package (v0.4.7; https://github.com/zhanghao-njmu/SCP) was used to visualize gene expression dynamics along inferred trajectories for granulosa cell subtypes. It was also used to compare these trajectories across species.

### Intercellular interaction analysis

Cell–cell communication across all species was inferred from ligand-receptor interactions using CellPhoneDB (v.5.0) [30]. Ligand-receptor interactions were identified across cell types based on a receptor library of human homologous genes. The average expression of each ligand-receptor pair was then compared across cell types to determine their cell type–specific expression profiles. Mean expression and statistical significance (*P*-value < 0.05) were calculated based on the normalized expression matrix obtained from Seurat.

## Results

### Single-cell transcriptional atlas of ovaries across species

**Fig. 1:**
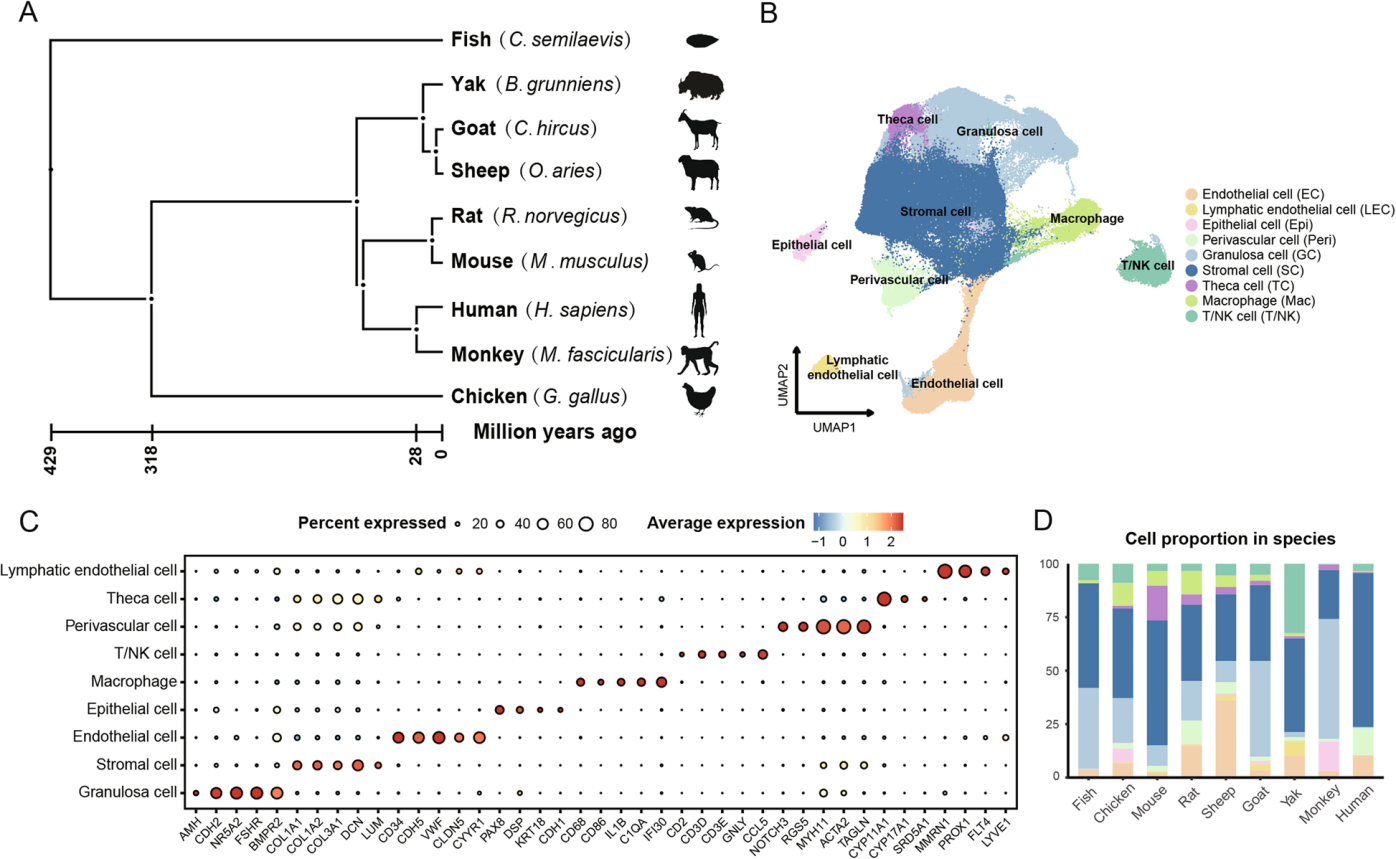
Ovarian cell type composition across species revealed by sc/snRNA-seq analysis. **A** Phylogenetic tree of nine vertebrate species based on TimeTree5 (https://timetree.org/). **B** UMAP of integrated scRNA-seq and snRNA-seq data for cell types. **C** Dot plot showing the expression of key marker genes (*x*-axis) across major cell types (*y*-axis) in all species. The depth of the color from light to dark and the size of the dots represent the average expression from low to high and the percentage of cells expressing the gene. **D** Proportion of each cell type in each species; colours correspond to cell types in Fig. 1B

In order to gain a comprehensive understanding of ovarian evolution, we conducted an in-depth analysis of the cellular composition and transcriptomic characteristics of ovaries across species. We collected published sc/snRNA-seq data of ovaries from nine vertebrate species, including Chinese tongue sole (*Cynoglossus semilaevis*), chicken (*Gallus gallus*), mouse (*Mus musculus*), rat (*Rattus norvegicus*), goat (*Capra hircus*), sheep (*Ovis aries*), yak (*Bos grunniens*), monkey (*Macaca fascicularis*), and human (*Homo sapiens*) (Fig. 1A). Based on scIB benchmarking results, RPCA was found to achieve the best integration performance for our datasets (Fig. S1C). Using canonical marker genes [14, 31–36] for cell type annotation, a total of 186,748 cells were classified into nine major cell types: endothelial cells (*CD34*, *CDH5*, *VWF*), lymphatic endothelial cells (*MMRN1*, *PROX1*, *FLT4*), epithelial cells (*PAX8*, *DSP*, *KRT18*), perivascular cells (*TAGLN*, *RGS5*, *ACTA2*), granulosa cells (*AMH*, *CDH2*, *FSHR*), stromal cells (*DCN*, *LUM*, *COL1A1*), theca cells (*CYP11A1*, *CYP17A1*), macrophages (*CD68*, *C1QC*, *IFI30*) and T/NK cells (*CCL5*, *CD3D*, *CD3E*) (Fig. 1B and C).

To explore cross-species cell type similarity, we used the AUROC score generated by MetaNeighbor to cluster the identified cell types. Overall, same cell types from different species tended to cluster together. Notably, macrophages and NK/T cells exhibited the highest similarity across species. However, the gene expression profiles of the remaining seven cell types were relatively conserved among species. Furthermore, cell-type similarity between fish and other species was comparatively low (Fig. S1A). We also assessed cell type similarity between snRNA-seq datasets (goats and monkey) and scRNA-seq datasets (other species), which showed generally high correlation despite differences in sequencing methods (Fig. S1B).

When examining ovarian cell types across all species, stromal cells were found to be widely distributed, whereas the proportions of other cell types varied among species (Fig. S1D). Overall, stromal cells constituted the largest proportion of ovarian cells, followed by granulosa cells in all species (Fig. 1D). Theca cells accounted for 16% of ovarian cells in mice but only 0.4% in humans. Interestingly, T/NK cells exhibited a high proportion in yak, comprising 32% of the total ovarian cells. Collectively, our atlas captures the majority of ovarian cell types across species, allowing us to directly compare ovarian cell types among vertebrates and to gain insights into the evolution and development of the vertebrate ovary.

### Identification of granulosa cell subtypes across species

**Fig. 2:**
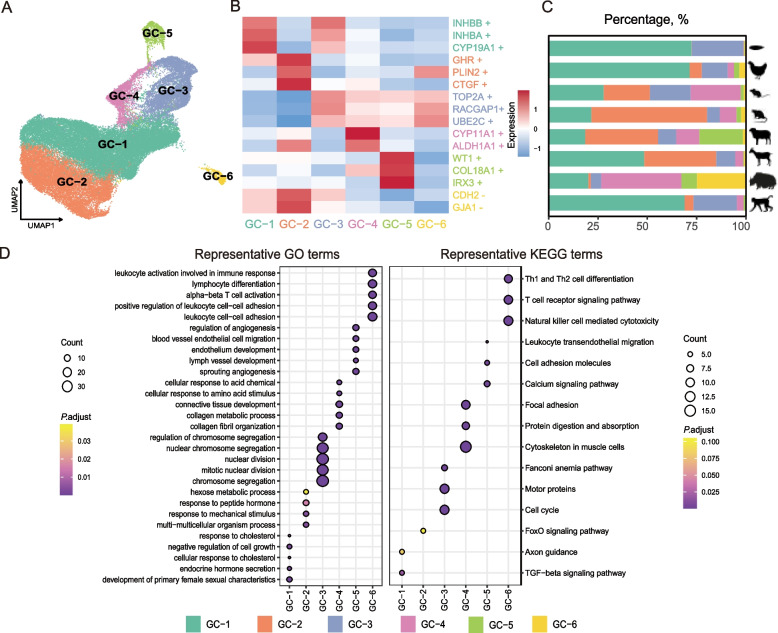
Granulosa cell subtype identification across species. **A** UMAP visualization of granulosa cells after dimensionality reduction, colored by cell subtypes. **B** Heatmap showing marker gene expression across granulosa cell sub-clusters, where "+" represents high expression and "–" represents low expression. **C** Stacked bargraphs depicting the composition of granulosa cell subclusters across eight species. **D** Top 100 Gene Ontology (GO) and Kyoto Encyclopedia of Genes and Genomes (KEGG) enrichment results, with color gradients ranging from purple (less significant) to yellow (more significant)

Granulosa cells are key ovarian components, playing essential roles in oocyte development, hormone synthesis, and reproductive regulation [37]. Granulosa cells from eight species were further classified into six cell subtypes, with their relative proportions varying among species (Fig. 2A and C). For example, GC-2 accounted for 0.17% of granulosa cells in fish and 1.25% in yak, whereas GC-6 represented about 24% of granulosa cells in yak (Fig. 2C). We also performed a downsampling analysis of granulosa cells, and found that the subtypes identified after downsampling were similar to the original subtypes, supporting the robustness and accuracy of our cell type assignments (Fig. S2A).

To explore the functional characteristics of each granulosa cell subtype, we performed GO and KEGG enrichment analyses based on the top 100 DEGs of each subtype (Fig. 2D, Table S4). The GC-5 cluster, characterized by high expression of *WT1* and *COL18A1* (Fig. 2B), was enriched for key pathways such as cell adhesion molecules and calcium signaling pathways (Fig. 2D). Based on marker gene expression and functional enrichment results, GC-5 was identified as early granulosa cells [34, 38]. DNA replication and cell proliferation-associated genes, such as *TOP2A* and *RACGAP1* [36], were highly expressed in the GC-3, which was enriched in pathways related to the cell cycle, chromosome separation, and mitotic division, indicating that GC-3 represents a proliferative granulosa cell subtype (Fig. 2B and D). GC-1 showed high expression of *INHBB*, *INHBA*, and *CYP19A1*, and was enriched in pathways related to cholesterol response, endocrine hormone secretion, female sexual development, and the TGF-β signaling pathway (Fig. 2B and D), suggesting that GC-1 corresponds to cumulus granulosa cells [39]. GC-2 was characterized by elevated expression of *GHR*, *PIK3IP1*, *PLIN2*, and *S100A6* (Fig. 2B and Fig. S2B). As *GHR* and *PIK3IP1* are commonly associated with atretic follicles, and *PLIN2* and *S100A6* with luteal cells, these expression patterns suggest that GC-2 represents an atretic or luteal-related granulosa cell population [36, 39]. GC-4 expressed *CYP11A1* and *ALDH1A1*, and was enriched in pathways related to connective tissue development and collagen organization, consistent with features of mural granulosa cells [40] (Fig. 2B). GC-6 exhibited low *GJA1* and *CDH2* expression, a pattern associated with atretic follicles [22]. In addition, GC-6 showed high expression of *PLIN2* and *S100A6*, suggesting that this cluster may represent a luteal-like cell population [36, 39]. We further examined the expression of marker genes of granulosa cell subtypes across species, and observed consistent patterns, supporting their cross-species applicability (Fig. S2B).

**Fig. 3:**
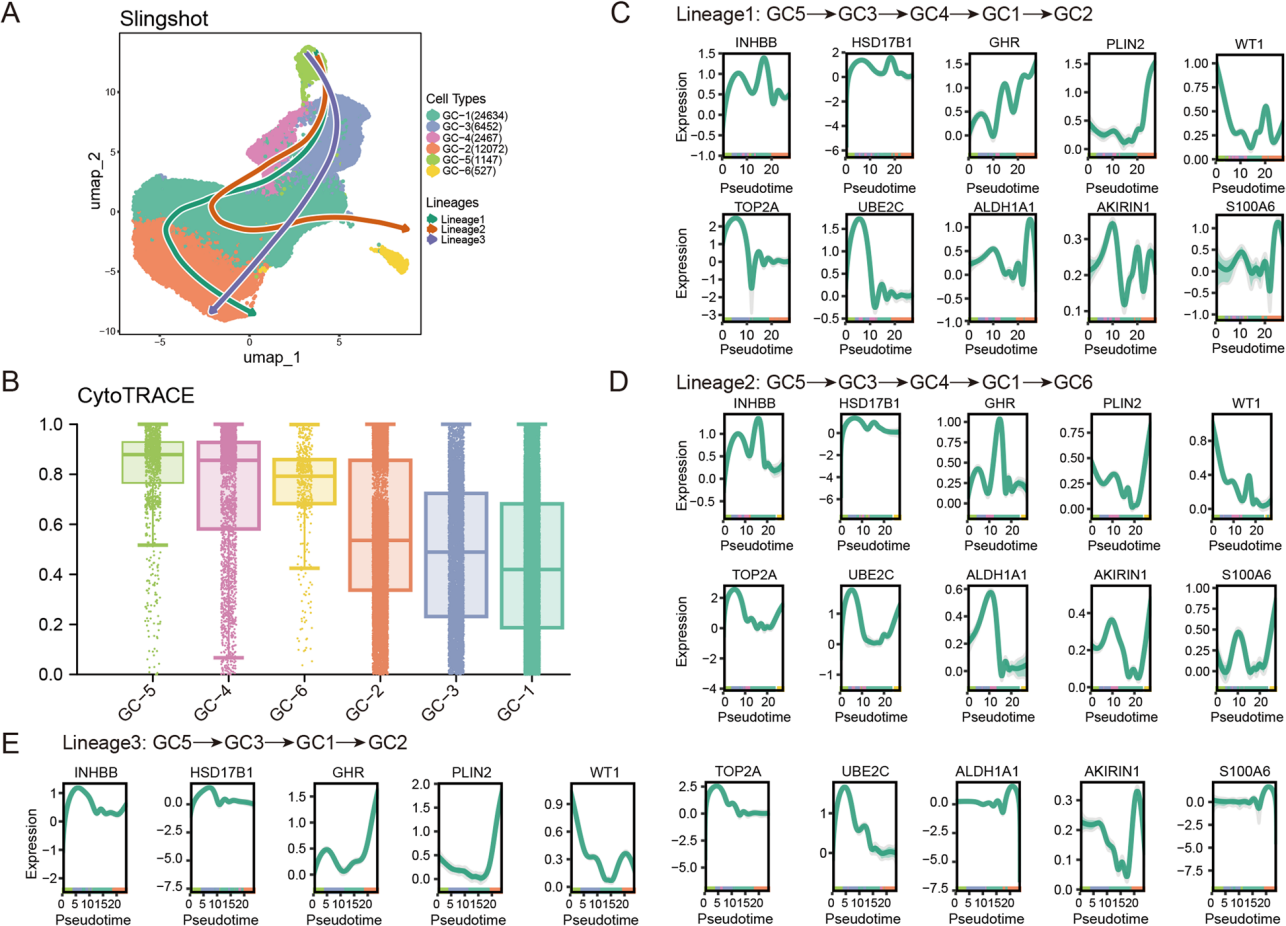
Granulosa cell differentiation trajectories and dynamics of key marker genes. **A** UMAP plot of Slingshot-derived granulosa cell trajectories. **B** Boxplot of CytoTRACE scores across granulosa cell subtypes. *X*-axis: granulosa cell subtypes; *Y*-axis: CytoTRACE score, indicating differentiation potential (higher scores represent less differentiated cells). **C–E** Dynamic expression of ten representative marker genes along pseudotime in Lineage 1 (**C**), Lineage 2 (**D**), and Lineage 3 (**E**)

Pseudotime trajectories of granulosa cells were constructed using Slingshot to investigate their developmental dynamics and transcriptional changes across species (Fig. 3A). CytoTRACE analysis identified GC-5 as the putative developmental starting point of granulosa cells (Fig. 3B). Based on this result, Slingshot revealed three distinct differentiation trajectories, all originating from GC-5 and representing three divergent granulosa cell lineages (Fig. 3A). Lineage 1 exhibited a stepwise progression through GC-3, GC-4, and GC-1, ultimately terminating at GC-2. In contrast, Lineage 2 followed a more direct differentiation route via GC-3 and GC-1 and converged on GC-2, whereas Lineage 3 progressed through GC-3, GC-4, and GC-1 before reaching GC-6. We next examined the overall expression dynamics of ten representative marker genes along the three inferred trajectories (Fig. 3C–E). Consistent with cell subtype identities, subtype-specific marker genes showed stage-dependent upregulation, with *INHBB* and *HSD17B1* displaying higher expression in GC-1, whereas *GHR* and *PLIN2* were more highly expressed in GC-2.

**Fig. 4:**
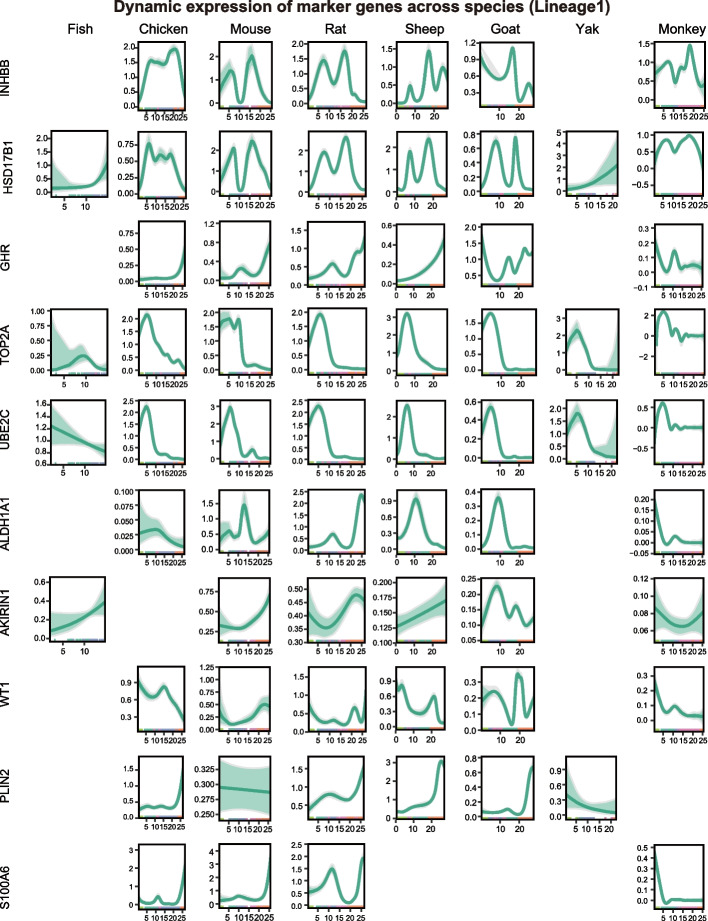
Cross-species dynamic expression of Lineage 1 marker genes along pseudotime in GCs

This analysis was further extended to a cross-species context. In Lineage 1 (Fig. 4), the expression dynamics of *TOP2A* and *UBE2C* were highly conserved across species, supporting evolutionary conservation of the meiotic stage in granulosa cell development. *INHBB*, *GHR*, and *WT1* displayed largely similar trends among chicken, mouse, rat, sheep, goat, and monkey. By contrast, *ALDH1A1* showed consistent patterns in mouse, sheep, and goat but diverged in chicken, rat, and monkey. *PLIN2* expression was absent in monkey. *S100A6* was detected only in chicken, mouse, rat, and monkey; it exhibited a gradual increase and peaked at GC-2 in chicken, mouse, and rat, whereas an opposite decreasing trend was observed in monkey. We also performed cross-species analyses of gene expression dynamics along Lineage 2 and Lineage 3 (Figs. S3 and S4). Taken together, our analysis uncovers conserved yet adaptable developmental trajectories of granulosa cells, reflecting shared molecular mechanisms and species-specific regulatory features during vertebrate folliculogenesis.

### Evolutionary conservation and divergence of granulosa cells in vertebrates

**Fig. 5:**
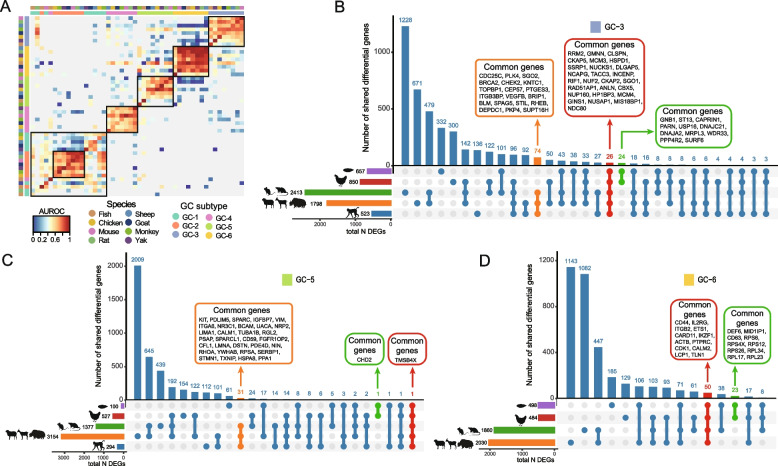
Granulosa cells exhibit variation across species. **A** Heatmap of "one_vs_best" MetaNeighbor scores for six granulosa cell subtypes across eight species. Cell types are labeled by both species and subtype. For each test dataset, cell subtype replicability scores (AUROCs) were calculated between the two nearest neighbors. **B–D** UpSet plots illustrating shared differentially expressed genes (DEGs) among five groups in GC-3 (**B**), GC-5 (**C**) and GC-6 (**D**)

We also assessed the conservation of granulosa cell subtypes across species (Fig. 5A). GC-6 showed the highest similarity across the eight species, followed by the GC-3 and GC-5. In GC-3, a total of 26 genes (e.g., *CKAP5*, *CKAP2*, *INCENP*, *MCM3*, and *GINS1*) were commonly shared among the five groups (fish, chicken, rodents, ruminants, and primates), indicating evolutionary conservation of the molecular machinery for granulosa cell proliferation across vertebrates (Fig. 5B). In contrast, differences in gene expression patterns of reproductive strategies revealed distinct regulatory preferences of granulosa cell function. For example, viviparous species exhibited enrichment of genes (e.g., *CHEK2*, *KNTC1*, *TOPBP1*, *CEP57*, *PTGES3*, and *ITGB3BP*) involved in DNA repair and signal transduction, whereas oviparous species showed enrichment in metabolic processes, represented by *GNB1*, *ST13*, *CAPRIN1*, *PARN*, and *USP16*. In GC-5, viviparity-associated genes included *IGFBP7*, *VIM*, *ITGA6*, *NR3C1*, and *BCAM*, whereas *CHD2* was the only gene related to oviparity (Fig. 5C). In GC-2, genes associated with viviparity included *IGF1R*, *STAT3*, *SOX4*, *NR4A1*, and *JAK1*, whereas genes related to oviparity included *SMCHD1*, *PFKL*, *PDS5B*, and *UCK1* (Fig. S5B). Some genes associated with viviparity and oviparity were also identified in GC-1, GC-4, and GC-6, respectively (Fig. 5D, Fig. S5A and C). *CDH2* [41], which encodes N-cadherin mediating cell–cell adhesion and structural integrity, was the only gene commonly shared across all five groups in GC-2, whereas *TMSB4X* [42], a regulator of actin dynamics that promotes granulosa cell migration and survival, was shared among all five groups in GC-5 (Fig. 5C and Fig. S5B).

Together, these findings suggest that granulosa cells retain a conserved molecular foundation for proliferation and differentiation, while acquiring species-specific transcriptional programs that adapt follicle development and hormonal regulation to distinct reproductive modes.

### Evolutionary conservation and divergence of stromal cells in vertebrates

**Fig. 6:**
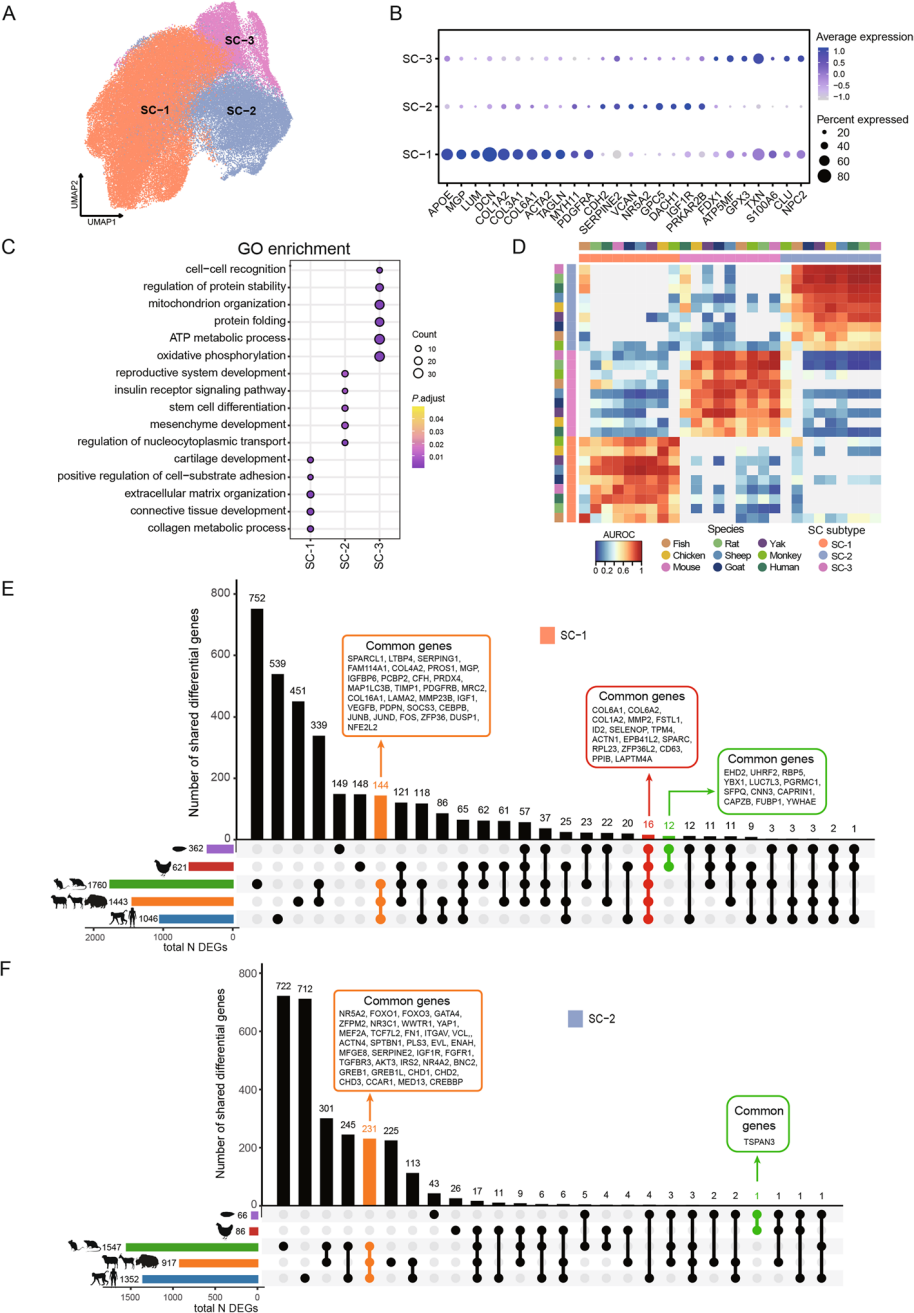
Stromal cell subtype identification across species. **A** UMAP visualization of stromal cells after dimensionality reduction, colored by cell subtypes. **B** Dot plot illustrating the expression patterns of selected signature genes across stromal cell subsets. Dot size represents the proportion of expressing cells, while color intensity reflects normalized expression levels. **C** Top 200 Gene Ontology (GO) enrichment results, with color gradients ranging from purple (less significant) to yellow (more significant). **D** Heatmap of "one_vs_best" MetaNeighbor scores for three stromal cell subtypes across nine species. Cell types are labeled by both species and subtype. For each test dataset, cell subtype replicability scores (AUROCs) were calculated between the two nearest neighbors. **E** and **F** UpSet plots illustrating shared differentially expressed genes (DEGs) among five groups in SC-1 (**E**), SC-2 (**F**)

Ovarian stromal cells provide structural support and secrete growth factors that regulate follicle development, ovulation, and tissue remodeling [43]. In our analysis, stromal cells accounted for the majority of all cell types and were further divided into three cell subtypes (Fig. 6A). SC-1, characterized by high expression of *LUM*, *MGP*, *COL1A2*, and *COL3A1*, was inferred to be a fibroblast-like stromal subtype [35, 36, 39] (Fig. 6B). GO enrichment analysis revealed significant enrichment in biological processes related to cartilage development, extracellular matrix organization, and connective tissue formation (Fig. 6C). SC-2, marked by *CDH2*, *SERPINE2*, *NR5A2*, and *IGF1R*, showed enrichment in pathways associated with reproductive system development and stem cell differentiation (Fig. 6B and C), suggesting that this subtype may be involved in processes supporting follicle development and hormone secretion. SC-3, marked by *FDX1*, *TXN*, and *S100A6*, exhibited enrichment in pathways related to cell–cell recognition, regulation of protein stability, and oxidative phosphorylation (Fig. 6B and C). In addition, analysis of stromal cell marker genes across different species revealed consistent expression patterns across species (Fig. S6A). For SC-1, *APOE*, *LUM*, *COL1A2*, and *COL6A1* were consistently expressed in most species. Similarly, SC-2 and SC-3 exhibited comparable expression patterns, characterized by *IGF1R* and *SERPINE2* in SC-2, and *FDX1* and *TXN* in SC-3.

MetaNeighbor analysis demonstrated that stromal cell subtypes exhibited a high degree of conservation across species (Fig. 6D). We then examined the genes shared by each of the three stromal subtypes across the five groups. In SC-1, a total of 16 genes were commonly shared, including *COL6A1*, *COL6A2*, *FSTL1*, and *ACTN1*. Notably, a total of 144 genes were highly conserved in viviparous species (e.g., *SPARCL1*, *LTBP4*, *SERPING1*, *PDGFRB*, and *MRC2*), whereas only 12 genes (including *EHD2*, *UHRF2*, *RBP5*, and *YBX1*) were shared in oviparous species (Fig. 6E). In SC-2, a total of 231 genes were associated with viviparity, compared to a single gene (*TSPAN3*) in oviparous species (Fig. 6F). In SC-3, a total of 32 genes were shared among viviparous species, whereas only one gene (*CD74*) was shared among oviparous species (Fig. S6B). Collectively, we revealed that ovarian stromal cells consist of functionally distinct subtypes with specific roles in follicle support, hormone regulation, and tissue organization, and that these subtypes are conserved across vertebrate evolution.

### Conservation and adaptation of intercellular communication in ovarian biology

**Fig. 7:**
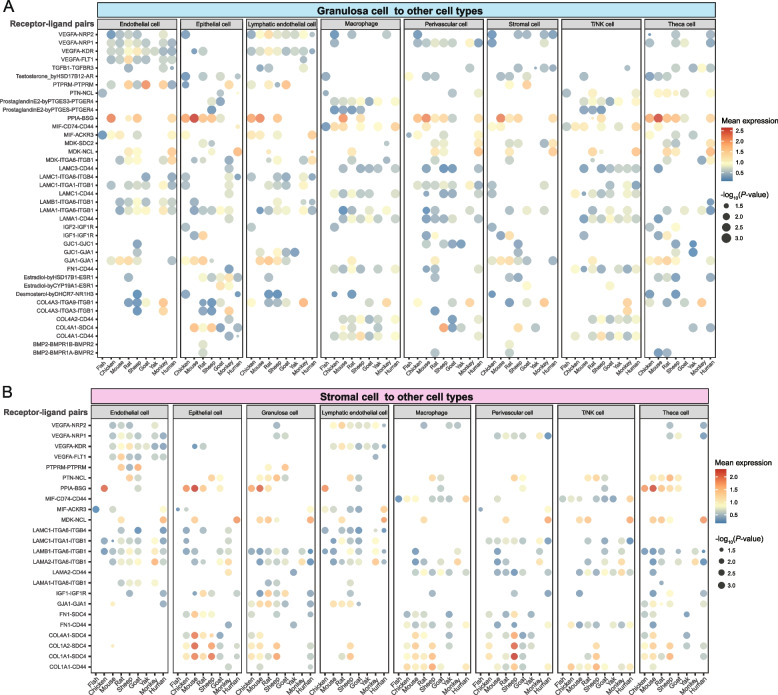
The interactions between granulosa or stromal cells and other ovarian cell types across species. **A** and** B** Dot plots depicting representative ligand–receptor interactions between granulosa cells and other ovarian cell types (**A**) and between stromal cells and other ovarian cell types (**B**). Interaction strength is shown by mean expression (color), and dot size reflects the statistical significance of each interaction (−log_10_ *P*-value)

To investigate the biological functions of granulosa cells and stromal cells, we used CellPhoneDB to analyze cell–cell interactions between granulosa cells and other ovarian cell types, as well as between stromal cells and other ovarian cell types. This analysis aimed to identify key ligand-receptor interaction pairs involved in ovarian intercellular communication (Fig. 7A and B).

Among interactions between granulosa cells and other ovarian cell types, several ligand-receptor pairs, such as COL4A1-CD44, GJA1-GJA1, LAMC1-ITGA1-ITGB1, and VEGFA-NRP2, showed conserved patterns across species, indicating their potential roles as fundamental signaling axes during follicular development (Fig. 7A). In contrast, GJA1-GJA1 exhibited a marked species-specific pattern, with high expression predominantly observed in rodents (mouse and rat), whereas lower expression levels were detected in sheep, goat, and human. Among collagen-integrin interactions, COL4A3-ITGA9-ITGB1 showed higher interaction in the monkey ovary than in other examined species. Estradiol-byHSD17B1-ESR1 and Estradiol-byCYP19A1-ESR1 highlight estradiol production via distinct enzymatic pathways in granulosa cells and the engagement of ESR1-expressing target cells. In stromal cell-mediated interactions with other ovarian cell types, several ligand-receptor pairs, such as COL1A1-SDC4, LAMB1-ITGA6-ITGB1, LAMA2-ITGA6-ITGB1, and VEGFA-NRP2, showed conserved patterns across species, mediating stromal cell adhesion, ECM remodeling, migration, and paracrine signaling (Fig. 7B). In interactions mediated by both granulosa and stromal cells, PPIA-BSG showed the highest expression in mouse, while MDK-NCL was predominantly expressed in human (Fig. 7A and B). Overall, intercellular communication among ovarian cells exhibited both evolutionary conservation and species specificity, which reflects that ovarian development in different species may be governed by species-specific signaling pathways.

## Discussion

The ovary provides an ideal system for exploring the evolutionary diversification of reproductive strategies among vertebrates, given its central role in gametogenesis, endocrine regulation, and follicular development [44]. Here, we constructed a comprehensive ovarian cell atlas from nine representative vertebrates, including oviparous and viviparous species, with single-cell and single-nucleus transcriptomic resolution. Our analysis revealed both conservation and lineage-specific innovation in ovarian cell types, highlighting how cellular architecture and communication networks have diversified during ovarian evolution in vertebrates.

We found that major somatic cell types, including endothelial, immune, stromal, and perivascular cells, exhibited strong transcriptional conservation, suggesting maintenance of fundamental ovarian homeostatic functions. In contrast, granulosa cells showed marked evolutionary divergence, reflecting their central roles in follicle development, steroidogenesis, and tissue remodeling [37]. Cross-species comparisons revealed pronounced variation in ovarian cell type composition: stromal cells predominated across vertebrates, whereas theca cells were relatively enriched in mice but nearly absent in humans, and T/NK cells displayed remarkable expansion in yak. These differences likely reflect species-specific reproductive strategies shaped by variation in follicle hierarchy, oocyte growth rates, seasonal breeding patterns, and the presence or absence of luteal phase biology between oviparous and viviparous lineages.

Granulosa cells were subdivided into transcriptionally distinct subtypes corresponding to different developmental and functional states. Core proliferative genes such as *CKAP5*, *CHEK2*, and *GINS1* were conserved across species, suggesting a shared molecular program for follicle growth and oocyte support [45–47]. In contrast, viviparous species exhibited upregulation of signaling and cell-cycle regulators (e.g., *CHEK2*, *ITGB3BP*, *IGFBP7*), consistent with enhanced requirements for sustained follicular development, luteal maintenance, and coordination of complex follicle hierarchies in internal fertilization [48]. *IGFBP7*, previously shown to modulate granulosa cell differentiation and steroidogenesis [48], may contribute to species-specific regulation of granulosa cell maturation. In oviparous species, the enrichment of RNA-processing and metabolic genes (e.g., *GNB1*, *CAPRIN1*, *PARN*) [49–51] implied adaptive optimization of rapid follicle turnover and yolk-dependent oocyte maturation.

Stromal cells, which represented the most abundant ovarian population, displayed conserved transcriptional signatures (e.g., *COL6A1*, *FSTL1*, *PDGFRB*) associated with extracellular matrix organization and structural support [52]. Collagen- and laminin-mediated adhesion, likely involving integrin receptors (e.g., *ITGA1*, *ITGB1*), represents a conserved signaling module essential for ovarian architecture and follicular integrity [53].

Evolutionary changes in the ovary are also reflected in intercellular communication networks, which combine deeply conserved signaling axes with lineage-specific regulatory adaptations. Ligand-receptor pairs such as COL4A1-CD44, mediating ECM–cell adhesion, and LAMC1–(ITGA1 + ITGB1), mediating ECM–integrin signaling, likely represent ancestral pathways that preserve follicle integrity and maintain ovarian architecture [53, 54]. Superimposed on this fundamental communication network, species-specific deployment of ovarian communication pathways appears to reflect differences in follicle developmental dynamics and post-ovulatory endocrine demands. GJA1-mediated gap junction signaling is prominently enriched in rodents, consistent with rapid oocyte growth and tightly synchronized folliculogenesis, whereas its reduced role in large mammals and humans may reflect slower follicle development and diminished reliance on gap-junctional coupling [55, 56]. Likewise, divergent utilization of PPIA–BSG in mouse and MDK–NCL in human reflects adaptive differences in ovarian regulatory mechanisms across vertebrates. In viviparous mammals such as humans, MDK–NCL signaling may support sustained stromal–granulosa communication associated with a prolonged luteal phase [57]. Together, these patterns indicate that conserved ovarian communication frameworks are selectively tuned to species-specific reproductive strategies.

However, our study has certain limitations. Differences in sample preparation strategies, sequencing depth, and sequencing modalities (scRNA-seq vs. snRNA-seq) across studies may influence the representation of specific cell populations, including variation in cell numbers among samples of the same species. To mitigate these effects, we maintained comparable sample conditions where possible, applied stringent quality control, and evaluated multiple data integration strategies using scIB for dataset integration and batch correction. Moreover, our analyses were primarily conducted at the cell-type level, which is expected to reduce the impact of such variation on cross-species comparisons.

## Conclusions

Together, these findings indicate that ovarian evolution is shaped by a balance between the conservation of essential cell lineages and the diversification of their transcriptional and signaling programs. This integrative cross-species framework not only provides molecular insights into the adaptive evolution of vertebrate reproduction but also establishes a valuable reference for future functional and evolutionary studies of ovarian biology.

## Supplementary Information

Additional file 1: Fig. S1. Cross-species and cross-platform validation of ovarian cell types. A Cross-species heatmap showing the mean area under the receiver operating characteristic (AUROC) scores from MetaNeighbor analysis, colored by ovarian cell subtypes. B AUROC-based cell type correlation between scRNA-seq and snRNA-seq datasets. C Benchmarking RPCA, CCA, Harmony integration, and only merged (without batch correction) using scIB. D UMAP plots showing the identified cell types and their composition across different species. Fig. S2. Downsampling-based validation of granulosa cell subtype clustering and cross-species marker gene profiling. A UMAP visualization of granulosa cell subtypes after repeated downsampling and AUROC-based correlation with the original subpopulations. B Dot plot showing the expression of key marker genes (*y*-axis) across GC subtypes in different species (*x*-axis). Dot color intensity represents average gene expression, and dot size indicates the percentage of cells expressing each gene. Fig. S3. Cross-species dynamic expression of Lineage 2 marker genes along pseudotime in GCs. Fig. S4. Cross-species dynamic expression of Lineage 3 marker genes along pseudotime in GCs. Fig. S5. Cross-species comparison of shared DEGs across five groups in GC-1, GC-2, and GC-4. A–C UpSet plots illustrating shared DEGs among five groups in GC-1 (A), GC-2 (B), and GC-4 (C). Fig. S6. Expression patterns and shared differentially expressed genes among SC subtypes across species. A Dot plot showing the expression of key marker genes (*y*-axis) across SC subtypes in different species (*x*-axis). Dot color intensity represents average gene expression, and dot size indicates the percentage of cells expressing each gene. B UpSet plot illustrating shared DEGs among five groups in SC-3.

Additional file 2: Table S1. Detailed description of the sc/snRNA-seq data used in this study. Table S2. QC information and Quality information. Table S3. One-to-one ortholog mapping between human and non-human species. Table S4. Top 100 genes specifically expressed in each granulosa cell subtype across species.

## Data Availability

All sequencing data analyzed in this study are publicly available from the China National Genebank (CNP0002319 and CNP0001469), ArrayExpress (E-MTAB-11491), the Genome Sequence Archive (CRA017114 and CRA008987), and the NCBI repositories (GSE207023, PRJNA1010653, GSE233801, GSE213989, and GSE255690). Any additional information is available from the corresponding author upon reasonable request.

## Abbreviations

AUROC: Area under the receiver operating characteristic curve
DEGs: Differentially expressed genes
GCs: Granulosa cells
GO: Gene Ontology
KEGG: Kyoto Encyclopedia of Genes and Genomes
RPCA: Reciprocal principal component analysis
SCs: Stromal cells
scRNA-seq: Single-cell RNA sequencing
snRNA-seq: Single-nucleus RNA sequencing
