# Supplementary material for: Cross-species single-cell transcriptomic analyses reveal evolutionary conservation and diversification of ovarian tissues
Source: J Anim Sci Biotechnol. 2026 Apr 14;17:67. doi: 10.1186/s40104-026-01383-1 (PMC13077819; doi:10.1186/s40104-026-01383-1)
Supplement: Supplementary file 1 — Additional file 1: Fig. S1. Cross-species and cross-platform validation of ovarian cell types. A Cross-species heatmap showing the mean area under the receiver operating characteristic (AUROC) scores from MetaNeighbor analysis, colored by ovarian cell subtypes. B AUROC-based cell type correlation between scRNA-seq and snRNA-seq datasets. C Benchmarking RPCA, CCA, Harmony integration, and only merged (without batch correction) using scIB. D UMAP plots showing the identified cell types and their composition across different species. Fig. S2. Downsampling-based validation of granulosa cell subtype clustering and cross-species marker gene profiling. A UMAP visualization of granulosa cell subtypes after repeated downsampling and AUROC-based correlation with the original subpopulations. B Dot plot showing the expression of key marker genes (y-axis) across GC subtypes in different species (x-axis). Dot color intensity represents average gene expression, and dot size indicates the percentage of cells expressing each gene. Fig. S3. Cross-species dynamic expression of Lineage 2 marker genes along pseudotime in GCs. Fig. S4. Cross-species dynamic expression of Lineage 3 marker genes along pseudotime in GCs. Fig. S5. Cross-species comparison of shared DEGs across five groups in GC-1, GC-2, and GC-4. A–C UpSet plots illustrating shared DEGs among five groups in GC-1 (A), GC-2 (B), and GC-4 (C). Fig. S6. Expression patterns and shared differentially expressed genes among SC subtypes across species. A Dot plot showing the expression of key marker genes (y-axis) across SC subtypes in different species (x-axis). Dot color intensity represents average gene expression, and dot size indicates the percentage of cells expressing each gene. B UpSet plot illustrating shared DEGs among five groups in SC-3. [file 40104_2026_1383_MOESM1_ESM.zip › Fig.S1.pdf]

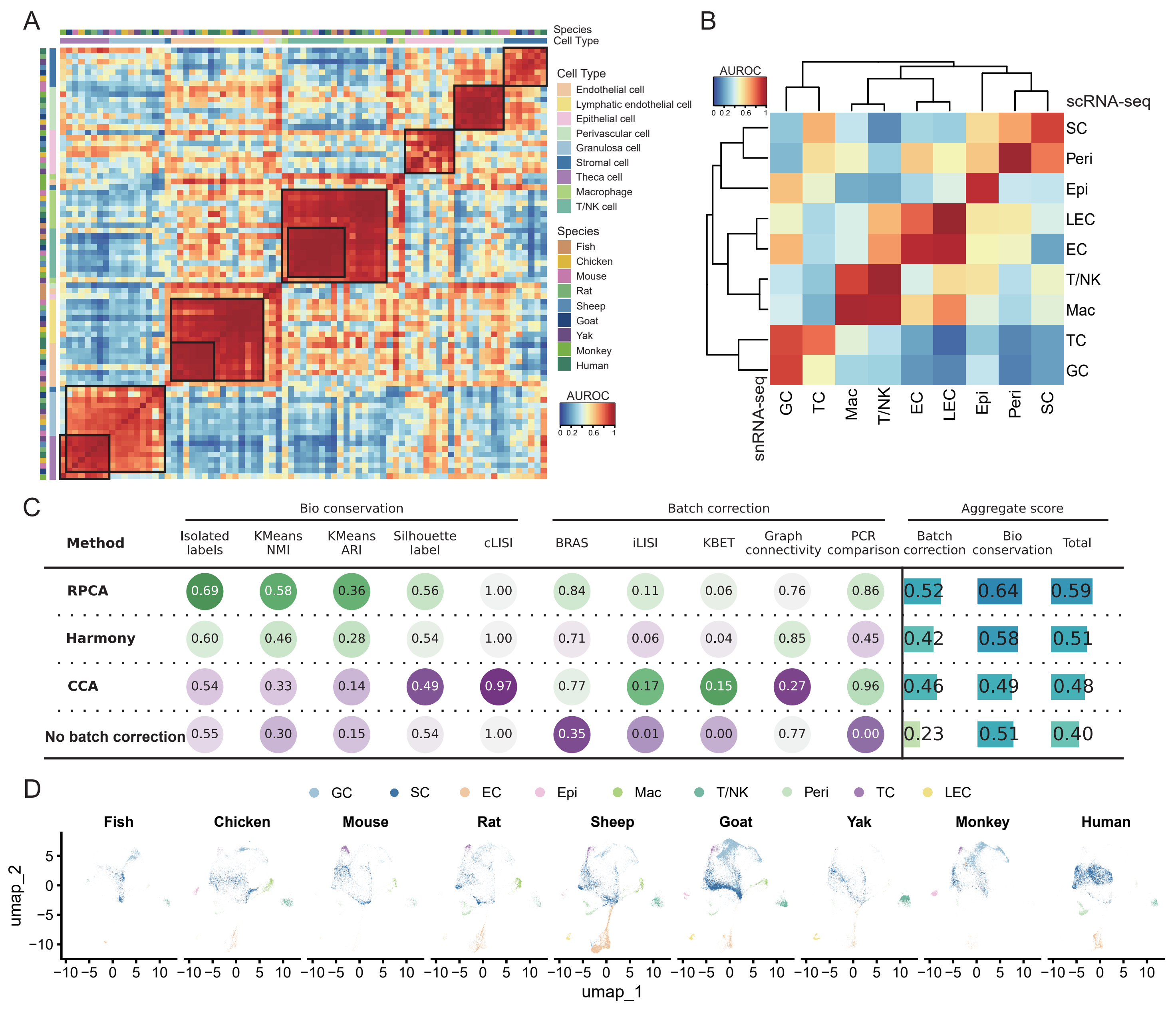
A
Species
Cell Type
Cell Type
Endothelial cell
Lymphatic endothelial cell
Epithelial cell
Perivascular cell
Granulosa cell
Stromal cell
Theca cell
Macrophage
T/NK cell
Species
Fish
Chicken
Mouse
Rat
Sheep
Goat
Yak
Monkey
Human
AUROC
0 0.2 0.6 1
B
AUROC
0 0.2 0.6 1
scRNA-seq
SC
Peri
Epi
LEC
EC
T/NK
Mac
TC
GC
snRNA-seq
GC TC Mac T/NK EC LEC Epi Peri SC
C
Bio conservation
Batch correction
Aggregate score
Method | Isolated labels | KMeans NMI | KMeans ARI | Silhouette label | cLISI | BRAS | iLISI | KBET | Graph connectivity | PCR comparison | Batch correction | Bio conservation | Total
RPCA 0.69 0.58 0.36 0.56 1.00 0.84 0.11 0.06 0.76 0.86 0.52 0.64 0.59
Harmony 0.60 0.46 0.28 0.54 1.00 0.71 0.06 0.04 0.85 0.45 0.42 0.58 0.51
CCA 0.54 0.33 0.14 0.49 0.97 0.77 0.17 0.15 0.27 0.96 0.46 0.49 0.48
No batch correction 0.55 0.30 0.15 0.54 1.00 0.35 0.01 0.00 0.77 0.00 0.23 0.51 0.40
D
GC SC EC Epi Mac T/NK Peri TC LEC
Fish Chicken Mouse Rat Sheep Goat Yak Monkey Human
umap_2
5
0
−5
−10
−10 −5 0 5 10
umap_1
