# Supplementary figures and images for: Cross-species single-cell transcriptomic analyses reveal evolutionary conservation and diversification of ovarian tissues
Source: J Anim Sci Biotechnol. 2026 Apr 14;17:67. doi: 10.1186/s40104-026-01383-1 (PMC13077819; doi:10.1186/s40104-026-01383-1)

A

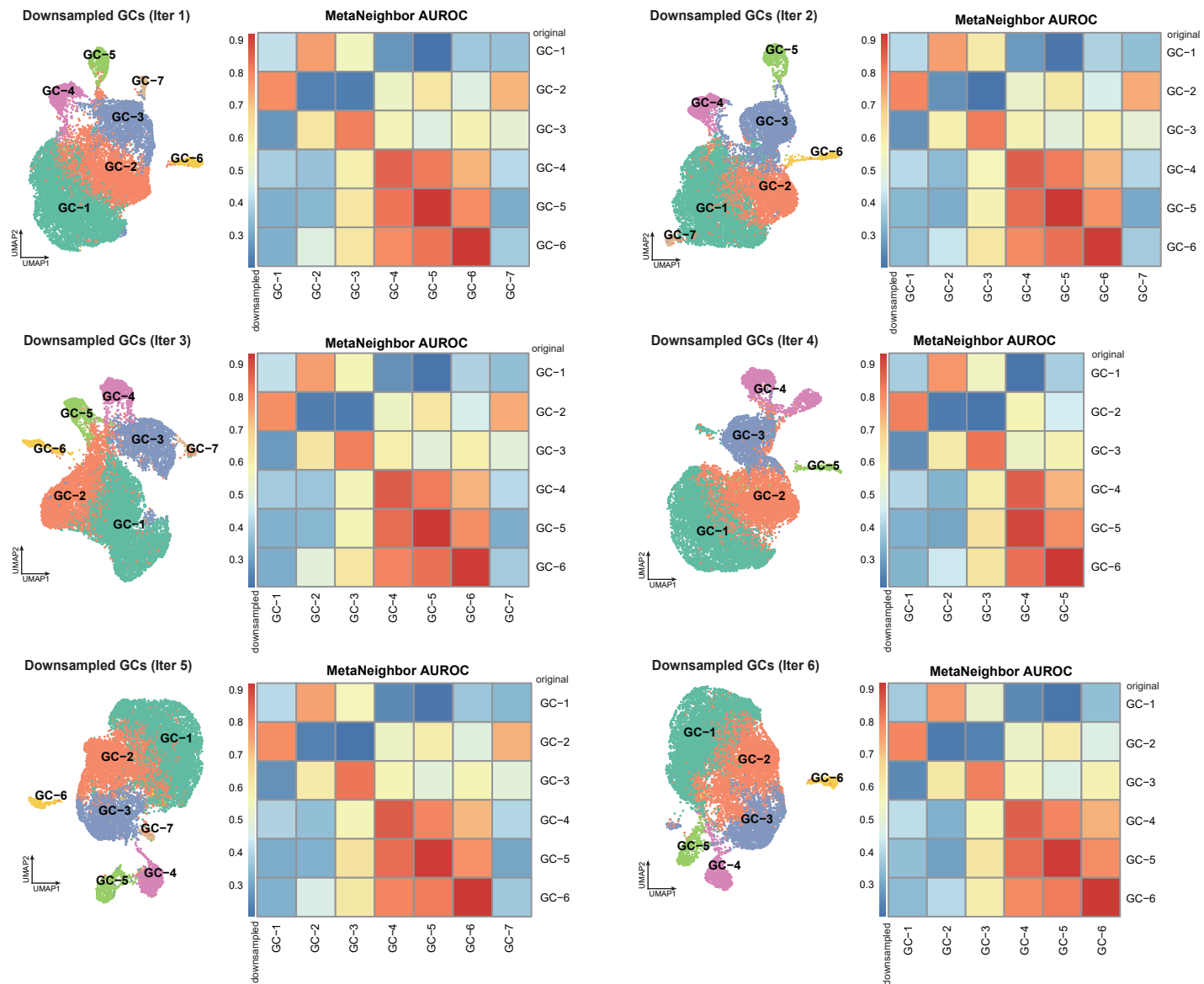

B

## Cross-species GC subtype markers

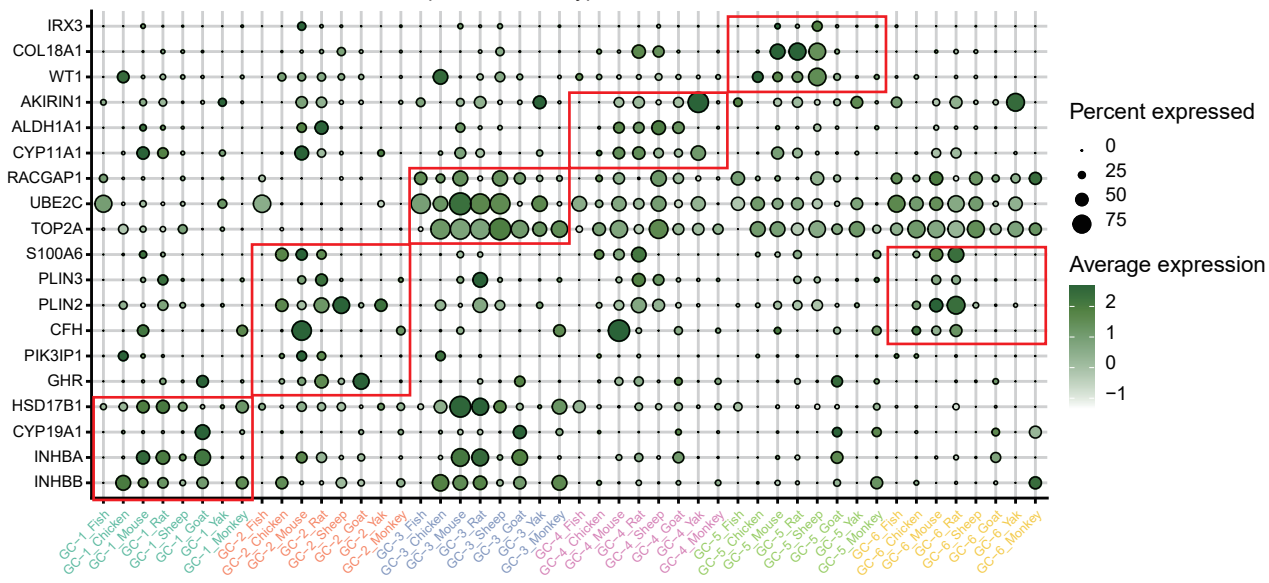

Supplement: Supplementary file 1 — Additional file 1: Fig. S1. Cross-species and cross-platform validation of ovarian cell types. A Cross-species heatmap showing the mean area under the receiver operating characteristic (AUROC) scores from MetaNeighbor analysis, colored by ovarian cell subtypes. B AUROC-based cell type correlation between scRNA-seq and snRNA-seq datasets. C Benchmarking RPCA, CCA, Harmony integration, and only merged (without batch correction) using scIB. D UMAP plots showing the identified cell types and their composition across different species. Fig. S2. Downsampling-based validation of granulosa cell subtype clustering and cross-species marker gene profiling. A UMAP visualization of granulosa cell subtypes after repeated downsampling and AUROC-based correlation with the original subpopulations. B Dot plot showing the expression of key marker genes (y-axis) across GC subtypes in different species (x-axis). Dot color intensity represents average gene expression, and dot size indicates the percentage of cells expressing each gene. Fig. S3. Cross-species dynamic expression of Lineage 2 marker genes along pseudotime in GCs. Fig. S4. Cross-species dynamic expression of Lineage 3 marker genes along pseudotime in GCs. Fig. S5. Cross-species comparison of shared DEGs across five groups in GC-1, GC-2, and GC-4. A–C UpSet plots illustrating shared DEGs among five groups in GC-1 (A), GC-2 (B), and GC-4 (C). Fig. S6. Expression patterns and shared differentially expressed genes among SC subtypes across species. A Dot plot showing the expression of key marker genes (y-axis) across SC subtypes in different species (x-axis). Dot color intensity represents average gene expression, and dot size indicates the percentage of cells expressing each gene. B UpSet plot illustrating shared DEGs among five groups in SC-3. [file 40104_2026_1383_MOESM1_ESM.zip › Fig.S2.pdf]

# Dynamic expression of marker genes across species (Lineage2)

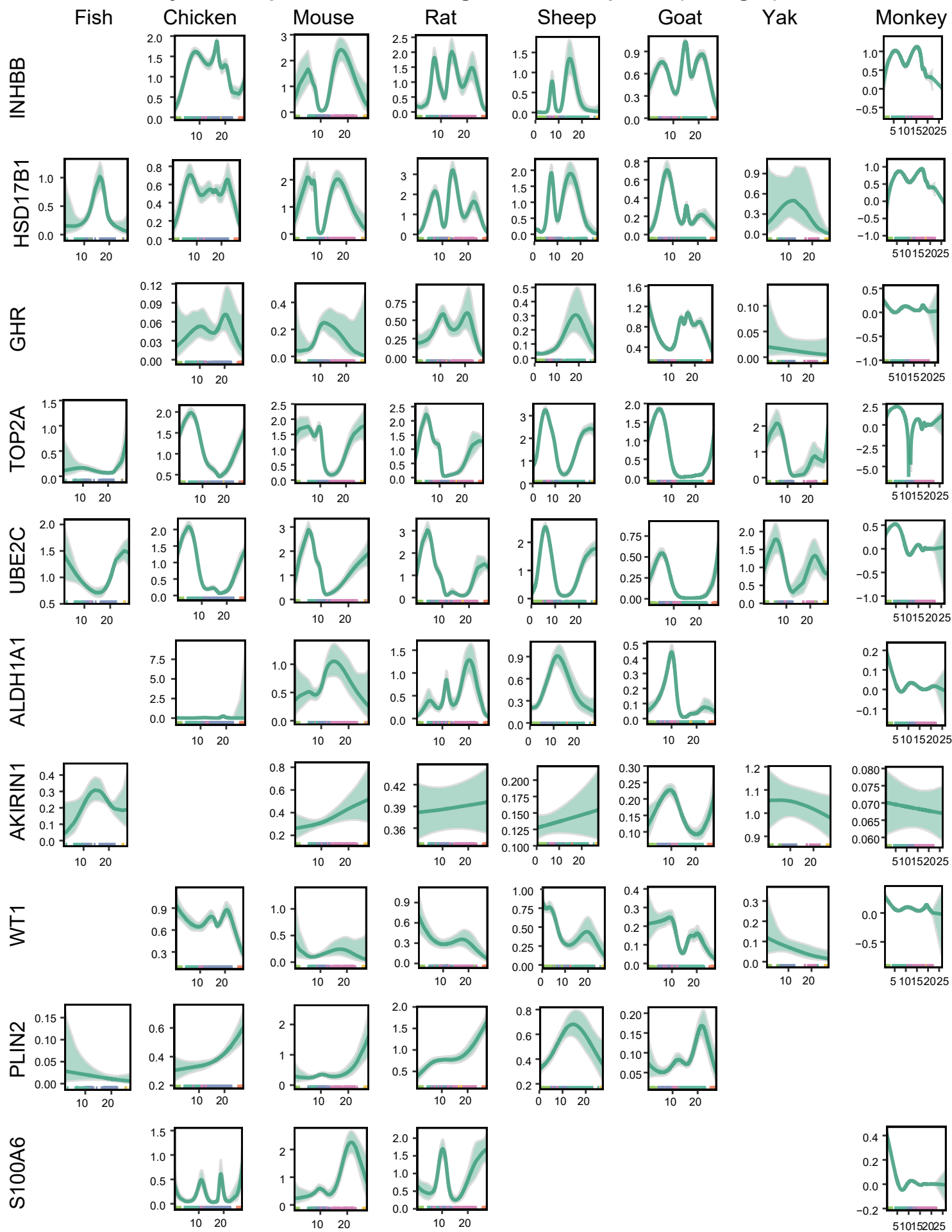

Supplement: Supplementary file 1 — Additional file 1: Fig. S1. Cross-species and cross-platform validation of ovarian cell types. A Cross-species heatmap showing the mean area under the receiver operating characteristic (AUROC) scores from MetaNeighbor analysis, colored by ovarian cell subtypes. B AUROC-based cell type correlation between scRNA-seq and snRNA-seq datasets. C Benchmarking RPCA, CCA, Harmony integration, and only merged (without batch correction) using scIB. D UMAP plots showing the identified cell types and their composition across different species. Fig. S2. Downsampling-based validation of granulosa cell subtype clustering and cross-species marker gene profiling. A UMAP visualization of granulosa cell subtypes after repeated downsampling and AUROC-based correlation with the original subpopulations. B Dot plot showing the expression of key marker genes (y-axis) across GC subtypes in different species (x-axis). Dot color intensity represents average gene expression, and dot size indicates the percentage of cells expressing each gene. Fig. S3. Cross-species dynamic expression of Lineage 2 marker genes along pseudotime in GCs. Fig. S4. Cross-species dynamic expression of Lineage 3 marker genes along pseudotime in GCs. Fig. S5. Cross-species comparison of shared DEGs across five groups in GC-1, GC-2, and GC-4. A–C UpSet plots illustrating shared DEGs among five groups in GC-1 (A), GC-2 (B), and GC-4 (C). Fig. S6. Expression patterns and shared differentially expressed genes among SC subtypes across species. A Dot plot showing the expression of key marker genes (y-axis) across SC subtypes in different species (x-axis). Dot color intensity represents average gene expression, and dot size indicates the percentage of cells expressing each gene. B UpSet plot illustrating shared DEGs among five groups in SC-3. [file 40104_2026_1383_MOESM1_ESM.zip › Fig.S3.pdf]

A

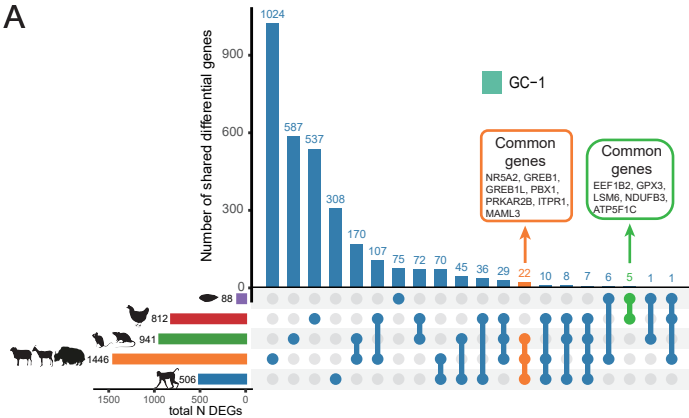

B

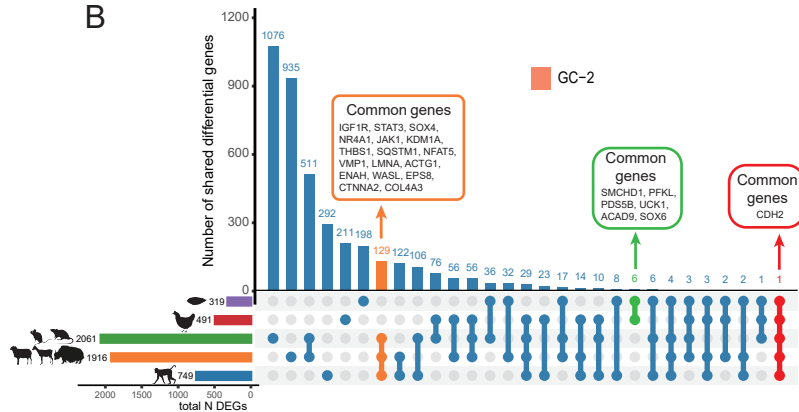

C

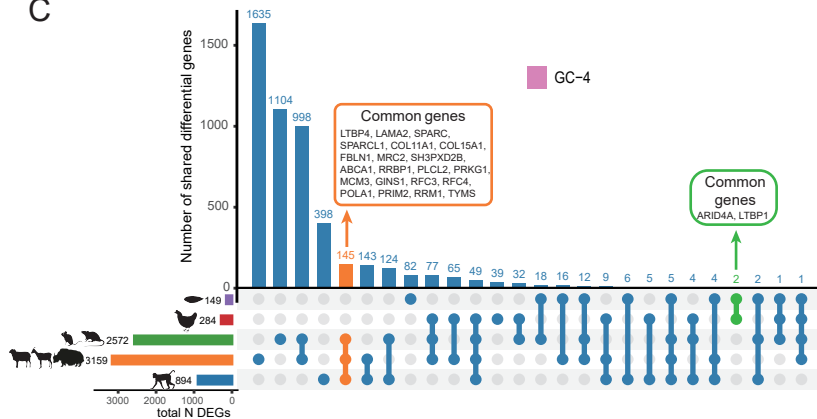

Supplement: Supplementary file 1 — Additional file 1: Fig. S1. Cross-species and cross-platform validation of ovarian cell types. A Cross-species heatmap showing the mean area under the receiver operating characteristic (AUROC) scores from MetaNeighbor analysis, colored by ovarian cell subtypes. B AUROC-based cell type correlation between scRNA-seq and snRNA-seq datasets. C Benchmarking RPCA, CCA, Harmony integration, and only merged (without batch correction) using scIB. D UMAP plots showing the identified cell types and their composition across different species. Fig. S2. Downsampling-based validation of granulosa cell subtype clustering and cross-species marker gene profiling. A UMAP visualization of granulosa cell subtypes after repeated downsampling and AUROC-based correlation with the original subpopulations. B Dot plot showing the expression of key marker genes (y-axis) across GC subtypes in different species (x-axis). Dot color intensity represents average gene expression, and dot size indicates the percentage of cells expressing each gene. Fig. S3. Cross-species dynamic expression of Lineage 2 marker genes along pseudotime in GCs. Fig. S4. Cross-species dynamic expression of Lineage 3 marker genes along pseudotime in GCs. Fig. S5. Cross-species comparison of shared DEGs across five groups in GC-1, GC-2, and GC-4. A–C UpSet plots illustrating shared DEGs among five groups in GC-1 (A), GC-2 (B), and GC-4 (C). Fig. S6. Expression patterns and shared differentially expressed genes among SC subtypes across species. A Dot plot showing the expression of key marker genes (y-axis) across SC subtypes in different species (x-axis). Dot color intensity represents average gene expression, and dot size indicates the percentage of cells expressing each gene. B UpSet plot illustrating shared DEGs among five groups in SC-3. [file 40104_2026_1383_MOESM1_ESM.zip › Fig.S5.pdf]

A

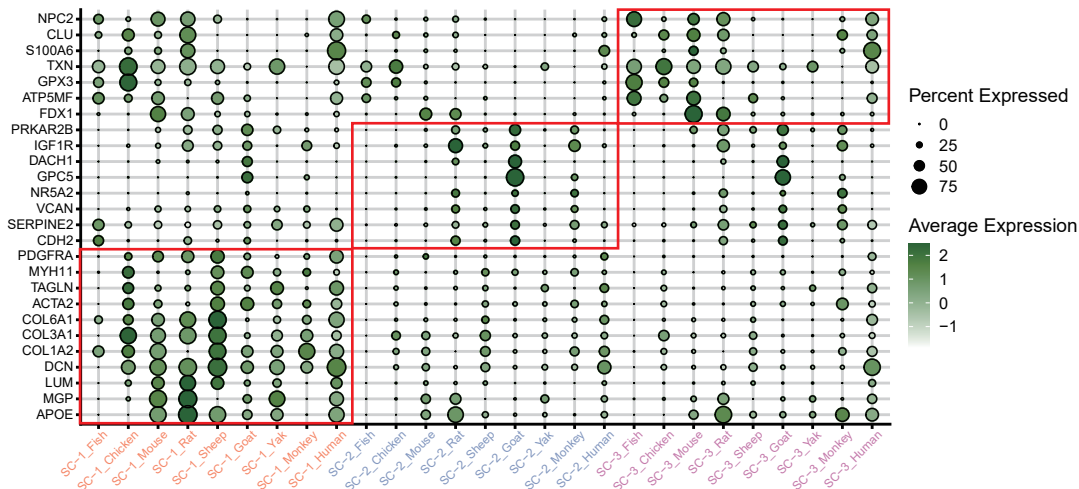

B

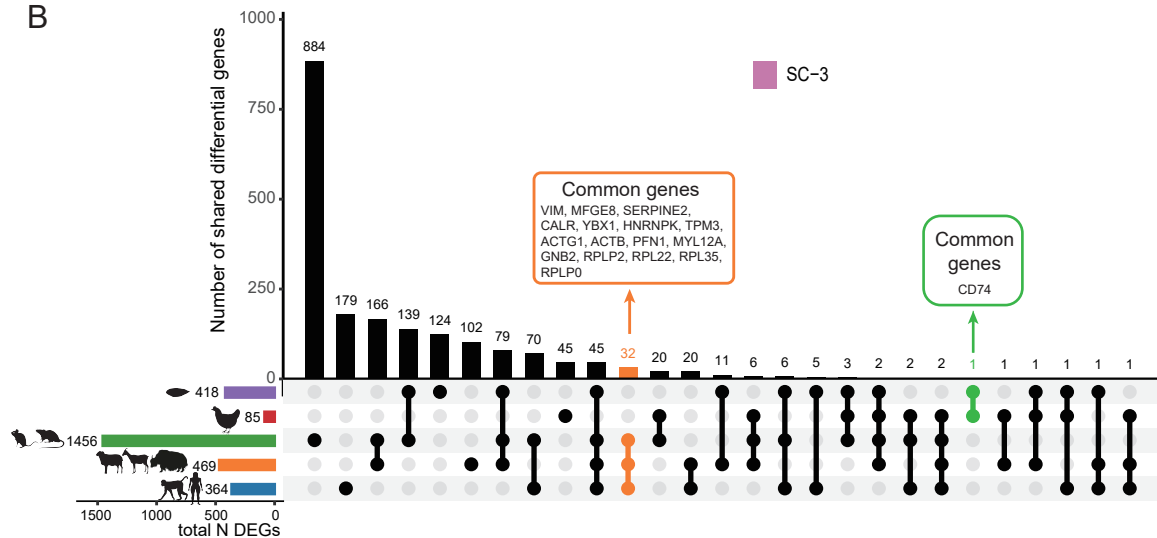

Supplement: Supplementary file 1 — Additional file 1: Fig. S1. Cross-species and cross-platform validation of ovarian cell types. A Cross-species heatmap showing the mean area under the receiver operating characteristic (AUROC) scores from MetaNeighbor analysis, colored by ovarian cell subtypes. B AUROC-based cell type correlation between scRNA-seq and snRNA-seq datasets. C Benchmarking RPCA, CCA, Harmony integration, and only merged (without batch correction) using scIB. D UMAP plots showing the identified cell types and their composition across different species. Fig. S2. Downsampling-based validation of granulosa cell subtype clustering and cross-species marker gene profiling. A UMAP visualization of granulosa cell subtypes after repeated downsampling and AUROC-based correlation with the original subpopulations. B Dot plot showing the expression of key marker genes (y-axis) across GC subtypes in different species (x-axis). Dot color intensity represents average gene expression, and dot size indicates the percentage of cells expressing each gene. Fig. S3. Cross-species dynamic expression of Lineage 2 marker genes along pseudotime in GCs. Fig. S4. Cross-species dynamic expression of Lineage 3 marker genes along pseudotime in GCs. Fig. S5. Cross-species comparison of shared DEGs across five groups in GC-1, GC-2, and GC-4. A–C UpSet plots illustrating shared DEGs among five groups in GC-1 (A), GC-2 (B), and GC-4 (C). Fig. S6. Expression patterns and shared differentially expressed genes among SC subtypes across species. A Dot plot showing the expression of key marker genes (y-axis) across SC subtypes in different species (x-axis). Dot color intensity represents average gene expression, and dot size indicates the percentage of cells expressing each gene. B UpSet plot illustrating shared DEGs among five groups in SC-3. [file 40104_2026_1383_MOESM1_ESM.zip › Fig.S6.pdf]
